# Association between antibiotic resistance and air pollution and climate factors in China: A multivariable spatial-temporal analysis

**DOI:** 10.1016/j.onehlt.2025.101204

**Published:** 2025-09-16

**Authors:** Bingsong Li, Xuemei Zhen, Jiangfeng Ouyang, Cecilia Stålsby Lundborg

**Affiliations:** a Department of Social Medicine and Health Management, School of Public Health, Cheeloo College of Medicine, Shandong University, Jinan 250012, China; b NHC Key Lab of Health Economics and Policy Research (Shandong University), Jinan 250012, China; c Center for Health Management and Policy Research, Shandong University (Shandong Provincial Key New Think Tank), Jinan 250012, China; d Zhucheng Center for Disease Control and Prevention, Zhucheng 262232, China; e Department of Global Public, Health, Karolinska Institutet, 17177 Stockholm, Sweden

**Keywords:** Carbapenem-resistant *Klebsiella pneumoniae*, Carbapenem-resistant *Escherichia coli*, Air pollution, China, GTWR

## Abstract

**Objective:**

In this study, we aimed to identify the spatiotemporal variation of antibiotic resistance and its relationship with environmental factors (air pollution and climate factors) in China.

**Method:**

The detection rate of *carbapenem-resistant Klebsiella pneumoniae* (CRKP) and *Escherichia coli* (CREC) in China from 2014 to 2022 was used to detect drug resistance. Air pollution factors include particulate matter and gaseous pollutants, and climate factors include temperature, precipitation and humidity. The spatial-temporal distributions and trend analyses were analyzed. Spatial autocorrelation analyses were performed to explore CRKP and CREC rates clustering. A geographically and temporally weighted regression model (GTWR) was adopted to analyze the relationship between antibiotic resistance rates and environmental factors.

**Result:**

We found that PM_2.5,_ O_3,_ annual average temperature and annual cumulative precipitation primarily had positive impacts on both CREC and CRKP rates. SO_2_, NO_2,_ CO and annual average humidity predominantly exerted negative impacts on both CREC and CRKP rates. Meanwhile, the influence of related factors on CREC and CRKP shows temporal and spatial heterogeneity. Both CREC and CRKP exhibited significant spatial aggregation, displaying High-High and Low-Low patterns. Trend analysis indicated an upward trend for both in the east-west direction and an inverted U-shape in the north-south direction.

**Conclusion:**

It is important to pay attention to the impact of air pollution and climate factors on antibiotic resistance.

## 1 Introduction

Antibiotic resistance poses a threat to global health and hinders progress in achieving sustainable development goals [1]. It was estimated that 4.95 million deaths were associated with antimicrobial resistance (AMR) in 2019, including 1.27 million deaths attributable to bacterial AMR [2]. If no urgent action is taken, there are an estimated 100 trillion USD in economic loss and 10 million deaths every year attributable to AMR around the globe by 2050, and there would be 20 trillion USD in economic burden and one million deaths in China [3,4].

*Klebsiella pneumoniae* and *Escherichia coli*, as two of the most well-known members of the *Enterobacter*, belonged to the top five bacterial pathogens responsible for infection-related deaths in 2019, each responsible for over 500,000 deaths globally [2]. Carbapenems are considered the last-line treatment option for infections caused by multidrug-resistant (MDR) bacteria. Increased use of carbapenem antibiotics has accelerated the emergence and global dissemination of carbapenem resistant *Klebsiella pneumoniae* (CRKP) and *Escherichia coli* (CREC), which have been declared priority AMR pathogens of public health importance [5]. Infections with CRKP and CREC are associated with increased morbidity and mortality compared with other pathogens, especially in patients with serious underlying disorders and immunocompromised individuals [[6], [7], [8]]. A review study showed that the global prevalence of hospital-acquired CRKP infections was 28.69 %, while it was more than 67 % in China [9]. A hospital study in China shows that the mortality rate of CREC infection has reached 29.1 % [10].

The One Health approach supports global health security by improving coordination, collaboration and communication at the human-animal-environment interface to address shared health threats such as antibiotic resistance [11]. The issue of antibiotic resistance is a cross-disciplinary health threat. Relevant research is no longer confined to human society but, based on the one health concept, explores the impact of animals and the environment on antibiotic resistance [11]. Current environmental issues such as air pollution and climate change may lead to a series of ecosystem changes, thereby increasing the risk of interaction at the human-environment interface and exposure to drug-resistant bacteria and genes [12,13]. In addition, the one health concept advocates cross-departmental and cross-disciplinary governance of antibiotic resistance, providing a foundation for multi-sectoral cooperation. A number of studies have shown that climate change factors, such as rising global temperatures, rainfall events, and air pollution factors like PM_2.5_, significantly affect the increase in antibiotic resistance [14]. For example, it was indicated that temperature could influence the development of CRKP [15]. Some studies also revealed that precipitation and humidity had an impact on antibiotic resistance including CREC [16,17]. PM_2.5_ air pollution had been found to contain a wide variety of antibiotic resistant bacteria and antibiotic resistant genes [18].

To the best of our knowledge, existing literature has explored antibiotic resistance and environmental factors [19,20]. However, research on the relationship between antibiotic resistance and air pollution factors remains inconclusive, and the relationship between it and climate factors also needs to be verified. There is a lack of long-term spatiotemporal studies on antibiotic resistance [21]. Traditional statistical methods often rely on regression and correlation analysis to examine the relationship between antibiotic resistance and influencing factors by comparing differences between various groups. However, these methods often overlook the spatial and temporal dimensions of the data. Changes in spatiotemporal dimensions are often not adequately captured by traditional statistical methods, and overlooking spatiotemporal changes may result in deviations in the findings. In particular, the antibiotic resistance rate, air pollution factors, and climate factors discussed in our study are closely linked to geographical space and vary over time [21]. Therefore, it is essential to consider the differences brought about by changes in time and spatial location. GTWR is a widely used model to explore the spatio-temporal variation between dependent and independent variables [22]. Environmental drivers of PM_2.5_ in China have been successfully investigated using the GTWR model [23]. At the same time, there are also relevant studies using the GTWR model to explore the socio-economic drivers of tuberculosis [24]. The GTWR model can capture the effects of space-time changes, making the results more reliable [25].

Therefore, we had three aims in this study. Firstly, explore the spatiotemporal distribution of CREC and CRKP. Secondly, the spatial aggregation of CREC and CRKP was explored through spatial autocorrelation analysis. Thirdly, this study aims to address the gap in the literature by exploring the quantitative spatial-temporal link between antibiotic resistance (CREC and CRKP) and environmental factors (air pollution and climatic factors) in China using the GTWR model.

## 2 Methods

### 2.1 Study design

This study adopts an ecological research design, uses provincial regions as observation units, and employs multivariate statistical techniques and spatio-temporal analysis methods, providing a framework for clarifying the driving mechanisms of cross-disciplinary issues related to antibiotic resistance.

#### 2.1.1 Dependent variable

Prevalence of antibiotic resistance was measured by the detection rates of CREC and CRKP in each year by province/region. The drug resistance rate of CREC and CRKP was obtained from the China Antimicrobial Resistance Surveillance System (CARSS). CARSS reports on the annual bacterial resistance monitoring situation in various provinces of China and continuously monitors the changes in bacterial resistance in each province. It has relatively complete provincial drug resistance monitoring data. CARSS totally covered 30 provinces and autonomous regions (Taiwan, Hong Kong, Macau, and Tibet were not included due to missing data during the study period of 2014–2022 (Fig. S1 in supplementary file)). The antibiotic susceptibility testing followed the guidelines and quality control requirements specified in the Clinical and Laboratory Standards Institute (CLSI) criteria. To avoid duplication, only the first episode of the same pathogen for one patient was included in each year, resulting in a total of 4,928,509 bacterial strains for analysis in 2022. In 2022, CARSS for analysis covered 1910 hospitals from all provinces and autonomous regions that participated, including 552 secondary hospitals (28.9 %) and 1358 tertiary hospitals (71.1 %). *E. coli* (29.1 %) and *K. pneumoniae* (21.2 %) are the two most frequently isolated gram-negative bacteria. The 9-year (2014–2022) panel dataset used in this study contained 270 observations records of annual prevalence of CREC and CRKP in the 30 provinces/regions of China (Table S1 in Supplementary file).

#### 2.1.2 Independent variables

Air pollution includes particulate matters (PM_2.5_ or PM_10_) and gaseous pollutants (O_3_, SO_2_, NO_2_, CO). A maximum 8-h 90th percentile concentration of O_3_, daily average 95th percentile concentration of CO, and annual average concertation of PM_2.5_, PM_10_, SO_2_, and No_2_ were extracted from China Statistic Yearbook. The selection of the above indicators all referred to the definition of exposure monitoring of air pollutants in the “Ambient Air Quality Standards” of China [26]. Additionally, climatic variables including annual average temperature, annual cumulative precipitation and annual average humidity were also obtained from China Statistic Yearbook (Table S1 in Supplementary file). Geographical information came from the National Geomatics Center of China (http://www.ngcc.cn/). Maps of China were produced using standard maps obtained from the Ministry of Natural Resources' standard map service website (http://bzdt.ch.mnr.gov.cn/) and authorized under the number GS (2023) 2767.

### 2.2 Statistical analysis

#### 2.2.1 Descriptive analysis

Temporal and spatial distributions of CREC and CRKP were analyzed by using Stata 17.0 and ArcGIS 10.8, respectively. For the spatial trend analysis, three-dimensional plot was used to observe east-west and north-south trends under a 90-degree projection. Thus, the rate of CREC (or CRKP) was considered as dependent variables (Z), and the geographical coordinates of longitude and latitude as independent variables (X and Y).

#### 2.2.2 Global spatial autocorrelation analysis

Global spatial autocorrelation analyses were performed in Geoda. Global Moran's Index (Moran's *I*) was used for global spatial autocorrelation analysis and the spatial distribution characteristics of CREC and CRKP rate were investigated from the overall level to determine whether spatial clustering existed in each province/region. After referring to relevant studies, we first constructed a spatial weight matrix based on the adjacency relationship [27]. The Global Moran's *I* formula is:

$$I=\frac{n}{\Sigma_{\mathrm{i}=1}^{n}\Sigma_{j=1}^{n}w_{i\dot{j}}}*\frac{\Sigma_{\mathrm{i}=1}^{n}\Sigma_{j=1}^{n}w_{\mathrm{ij}}z_{i}z_{j}}{\Sigma_{\mathrm{i}=1}^{n}z_{i}^{2}}$$

where n is the number of spatial units (provinces/regions), $w_{i\dot{j}}$ is the spatial weight between units i and $\dot{j}$, $z_{i}$ and $z_{j}$ are the differences between the attribute values for spatial units i and $\dot{j}$, respectively, and the mean of the attribute values across all spatial unit ($\bar{z}$). The formula for calculating the *Z*-statistic for global Moran's *I* is:

$$Z=\frac{I-E\left[I\right]}{\sqrt{v\left[I\right]}}$$

where $E\left[I\right]=-1/\left(n-1\right)$ and $V\left[I\right]=E\left[I^{2}\right]-E{\left[I\right]}^{2}$. If Z-statistic is greater than 1.96 or less than −1.96, this indicates that Global Moran's *I* is statistically significant at the 95 % confidence level. The Global Moran's *I* range from −1 to 1. If *I* > 0, this indicates positive spatial correlation, meaning that similar values tend to be clustered together in space. The larger the value, the stronger positive spatial correlation. If *I* < 0, this indicates negative spatial correlation, meaning that dissimilar values tend to be clustered together in space. The smaller the value, the stronger the negative spatial correlation. If *I* = 0, this suggests that there is no spatial autocorrelation, and the distribution of values is spatially random [24,28].

#### 2.2.3 Local spatial autocorrelation analysis

Local autocorrelation analysis builds upon global autocorrelation analysis to conduct a more detailed examination of specific clustered regions, which was performed using GeoDa. Local spatial autocorrelation was used to identify patterns of local clusters and their relationships with neighboring areas. The local Moran's *I* is calculated as follows:

$$I_{i}=\frac{x_{i}-\bar{X}}{s_{i}^{2}}\sum_{j=1,j\neq i}^{n}w_{i,j}\left(x_{j}-\bar{X}\right)$$

Where $x_{i}$ is the CREC rate (CRKP rate) for province(region) i, X is the mean of the CREC rate (CRKP rate), $w_{i,j}$ is the spatial weight between unit (province/region) i and j, and:

$$S_{i}^{2}=\frac{\Sigma_{j=1,j\neq n}^{n}{\left(\chi_{j}-\bar{X}\right)}^{2}}{n-1}-{\bar{X}}^{2}$$

With n is equal to the total number of the spatial units (provinces/regions).

The results of local spatial autocorrelation were categorized into four levels: high-high, low-high, high-low, and low-low, and were expressed using both Moran scatter plots and LISA cluster maps. High-High indicates that area with high drug resistance is surrounded by neighboring areas that also exhibit high values. Low-High indicates that area with low drug resistance surrounded by other areas with high values. Low-Low indicates that area with low drug resistance surrounded by other areas with low values. High-Low indicates that area with high drug resistance surrounded by other areas with low values. The High-High and Low-Low categories represent positive spatial autocorrelation, while the Low-High and High-Low categories represent negative spatial autocorrelation [29].

### 2.3 Influence factors analysis

#### 2.3.1 Multiple collinearity test

The influence factors included in the GTWR model were tested by variance inflation factor (VIF) to avoid the high collinearity between the independent variables affecting the regression analysis results [30]. The formula for calculating the VIF is:

$$\mathrm{VIF}=\frac{1}{\left(1-r^{2}\right)}$$

Where r^2^ is the coefficient of determination in the linear regression. The r^2^ reflects the percentage of change the variation in the dependent variable (Y) is explained by the independent variables (X). The larger the VIF, the greater the possibility of collinearity between explanatory variables. If all VIF values in a model fall within the range of 0 to 10, it is generally considered safe to proceed with the regression analysis without worrying about high collinearity [31]. In this study, we excluded PM_10_ due to high multicollinearity (VIF > 10) (Table S3 in Supplementary file).

#### 2.3.2 Model comparison

A total of four regression models including ordinary least square (OLS) model, geographically weighted regression (GWR) model, temporally weighted regression (TWR) model, and GTWR model were implemented to evaluate how the air pollution and climate factors are shaping the rates of CREC and CRKP in China. Unlike OLS model, which assumes that the spatial and temporal distribution of data is uniform and ignore the heterogeneity of data in space and time, the GTWR model captures the spatial-temporal heterogeneity of data by using spatial-temporal weights. Compared to GWR, which only accounts for spatial variation, and TWR, which only considers temporal variation, the GTWR model integrates both dimensions into a single framework to address the issue of spatiotemporal non-stationarity in data analysis [32]. We referred to existing studies and compared the coefficient of determination (R^2^), bandwidth and Akaike's information criterion (AICc) of the four models one by one [33]. After comprehensively considering the applicability of the data and three indicators, we selected GTWR as the optimal model. (Table S4 in Supplementary file). The R^2^ value of the GTWR model is higher than that of the OLS, TWR and GWR models, while the AICc value of the GTWR model is smaller than that of the OLS and TWR models, but slightly larger than that of the GWR model. The R^2^ of the GTWR model is the largest, indicating that its explanatory power is the best among the four models [33]. In terms of bandwidth, GTWR has a smaller bandwidth than GWR and TWR, indicating that it can capture the driving effect of influencing factors at a finer scale. In terms of the AICc value, GTWR is slightly higher than GWR, which indicates that the GTWR model is more complex than the GWR model due to its consideration of temporal and spatial heterogeneity. Considering the temporal and spatial heterogeneity of research data, while the GWR model only takes space into account and does not pay attention to temporal heterogeneity, the GTWR model is superior in data adaptation [34]. In summary, although the GTWR model increases the complexity to a certain extent, this study still believes that the GTWR model has better fitting effect in terms of most evaluation indicators and can better explain the driving effect of influencing factors from both time and space dimensions. It is worth noting that this study employed the GTWR model to explore the correlation between antibiotic resistance and environmental drivers, rather than a causal relationship.

#### 2.3.3 Geographically and temporally weighted regression (GTWR) model

The established GTWR model is as follows [22,32]:

$$Y_{i}\equiv \beta_{0}\left(U_{i},v_{i},t_{i}\right)+\Sigma_{k=1}^{m}\beta_{k}\left(U_{i},\nu_{i},t_{i}\right)X_{\mathrm{ik}}+\varepsilon_{i}$$

where ($U_{i},v_{i},t_{i}$) represents the spatial ($U_{i},v_{i}$) and temporal ($t_{i}$) coordinates of the observational unit. $\beta_{0}$($U_{i},v_{i},t_{i}$) is the regression constant for the *i*^th^ observational unit, and $\beta_{k}\left(U_{i},\nu_{i},t_{i}\right)$ denotes the regression coefficient of the *k*^th^ independent variable for the *i*^th^ observational unit. $X_{\mathrm{ik}}$ indicates the *k*^th^ independent variable for the *i*^th^ observational unit. $\varepsilon_{i}$ is the random error term, representing the random fluctuations in the dependent variable that the model cannot explain.

The regression coefficients in model fitting were calculated by the following formula:

$$\hat{\beta }\left(U_{i},v_{i},t_{i}\right)={\left[X^{T}w\left(U_{i},v_{i},t_{i}\right)X\right]}^{-1}X^{T}w\left(U_{i},v_{i},t_{i}\right)Y$$

where *W* (*u*_*i*_*,vi,t*_*i*_) is equal to diag (*α*_*i1*_*, α*_*i2*_*, α*_*i3*_*, …, α*_*in*_) and *n* is the count of research objects. The diagonal element *α*_*ij*_ (*1* *≤* *j* *≤* *n*) here is a function of temporal and spatial distance (*u,v,t*), and corresponds to the weight value when calibrating the weighted regression adjacent to the objection *i*. Therefore, the GTWR model relies on accurately specifying the temporal and spatial distance attenuation function *α*_*ij*_. To account for the size effects of different positions and times, an ellipsoidal coordinate system was employed to calculate the temporal and spatial interval between the regression point and the ambient measured data. In addition, based on a Gaussian distance attenuation function and Euclidean distance, a spatiotemporal weight matrix was also constructed [23]. In this study, an adaptive bandwidth selection method was employed, which determines both the bandwidth and model based on the modified Akaike Information Criterion(AICc) [27].

For the regression coefficients for independent variables, firstly, mean, standard deviation and quartile were calculated; then, for time heterogeneity analysis, we categorized these regression coefficients by year and constructed violin plots to show how these coefficients evolved over time; finally, in spatial heterogeneity analysis, we divided the coefficients by province, calculated the mean values for each province, and produced visual maps to illustrate the provincial disparities in the effects of these coefficients.

#### 2.3.4 Sensitivity analysis

To verify the robustness of the results, we conducted a set of sensitivity analyses. We adopt the global regression spatial error model to replace the local regression GTWR model. Meanwhile, since we did not control the corresponding socio-economic variables in the GTWR model, and considering that socio-economic variables also have a impact on the issue of antibiotic resistance, we included the logarithm of per capita GDP, the number of physicians per 10,000 population, and the number of hospital beds per 10,000 population in the spatial error model for control. We compared the R^2^ of the spatial error model and the GTWR model to compare their explanatory power. At the same time, compare the coefficients of the variables to verify the robustness of the results.

## 3 Results

### 3.1 Temporal-spatial distribution

**Fig. 1:**
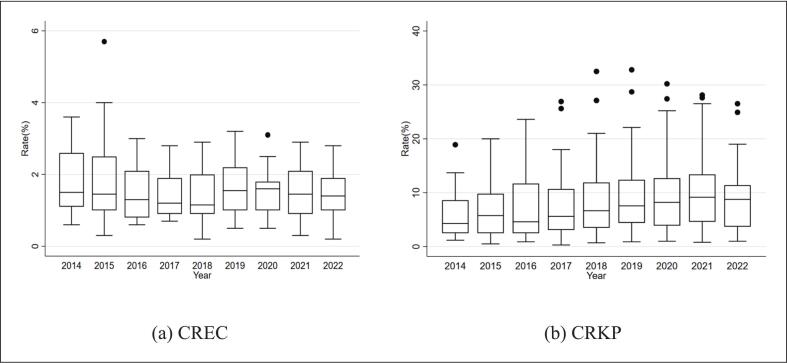
Temporal distribution of CREC and CRKP. Note: CREC: Carbapenem resistant *Escherichia coli*, CRKP: Carbapenem resistant *Klebsiella pneumoniae.*

From 2014 to 2022, the rate of CREC exhibited relative stability, with its median values consistently below 2 %. From 1.5 % in 2014 to 1.2 % in 2018, there was a certain downward trend, but it has started to rise in recent years, reaching 1.4 % in 2022. On the contrary, during the same period, the ratio of CRKP continued to rise, with the median increasing from 4.3 % in 2014 to 9.3 % in 2022. (Fig. 1).

**Fig. 2:**
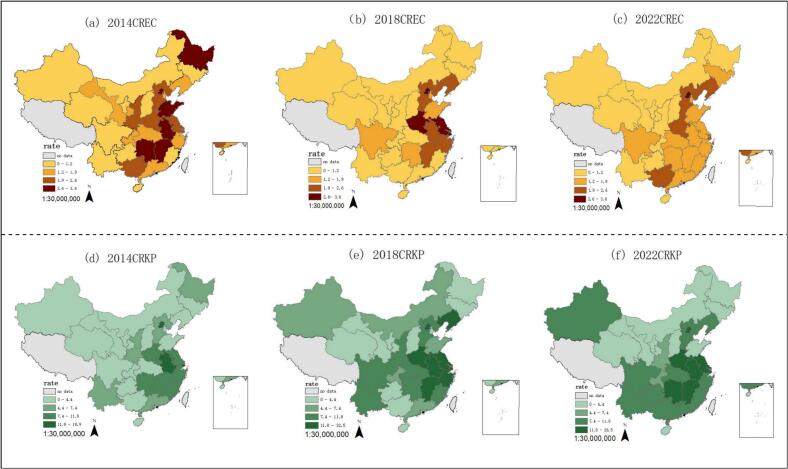
Spatial distribution of CREC and CRKP. Note: CREC: Carbapenem resistant *Escherichia coli*, CRKP: Carbapenem resistant *Klebsiella pneumoniae.*

In 2014, the CREC rates were notably high in the provinces of Heilongjiang, Hunan, Jiangxi, Anhui, and Shandong, however, by the years 2018 and 2022, there was a general decrease. Overall, the rates of CREC were higher in the eastern regions compared to the western regions, and it is also more pronounced in the northern areas than in the southern areas. In addition, it was noted that CRKP rates consistently remained high in southern China and Xinjiang Uyghur Autonomous Region (Fig. 2).

**Fig. 3:**
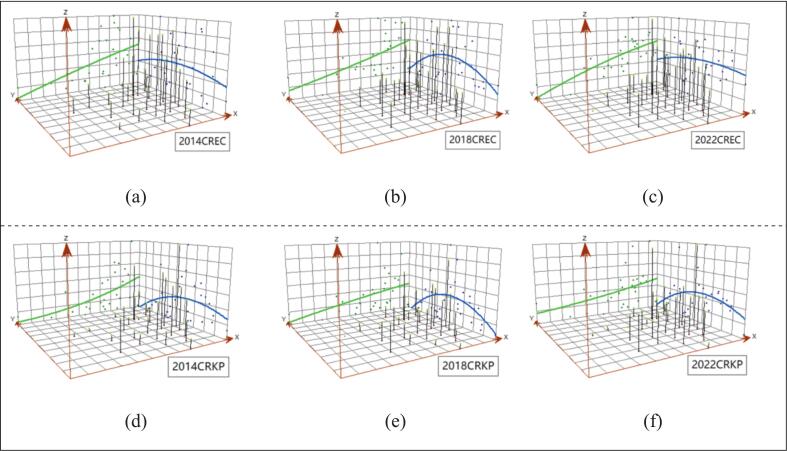
Spatial trends of CREC and CRKP. Note: CREC: Carbapenem resistant *Escherichia coli*, CRKP: Carbapenem resistant *Klebsiella pneumoniae.*

For the trend analysis, it showed that the overall trend of CREC and CRKP rates were largely consistent across the years, with a discernible upward trajectory both in the eastward and westward directions; that is, proximity to the eastern regions correlates with increased resistance rates. Additionally, there is a notable inverted U-shaped distribution when comparing northern and southern regions, peaking in the center and dipping at the extremities, which implied higher rates of CREC and CRKP in the central regions (Fig. 3).

### 3.2 Global spatial correlation patterns

**Fig. 4:**
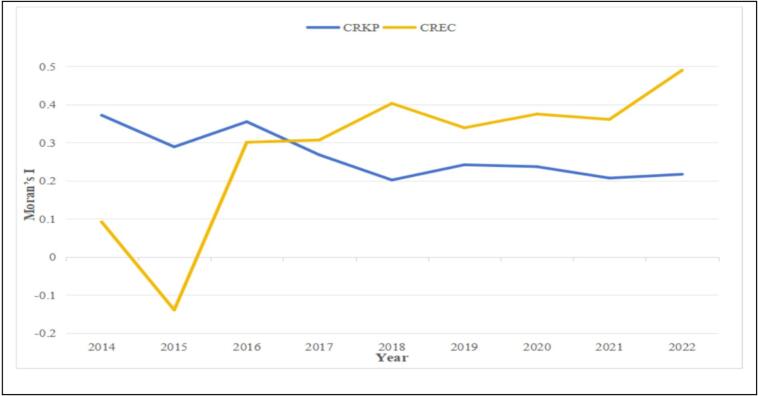
Global Moran Index of CKEC and CRKP during 2014–2022. Note: CREC: Carbapenem resistant *Escherichia coli*, CRKP: Carbapenem resistant *Klebsiella pneumoniae.*

From 2014 to 2022, the Global Moran's *I* indicated significantly spatial clustering of CREC and CRKP rates in China. Global Moran's I for CREC rate experienced a substantial rise, climbing from 0.301(*p* < 0.012) in 2016 to 0.49(*p* < 0.001) by 2022. This significant growth denoted an increasingly prominent tendency spatial clustering among provinces, coupled with an enhanced degree of spatial clustering. The Global Moran's *I* for CRKP showed a statistically significant and positive spatial correlation, indicating a persistent pattern of spatial clustering (Fig. 4 and Table S2 in Supplementary file).

### 3.3 Local spatial correlation patterns

**Fig. 5:**
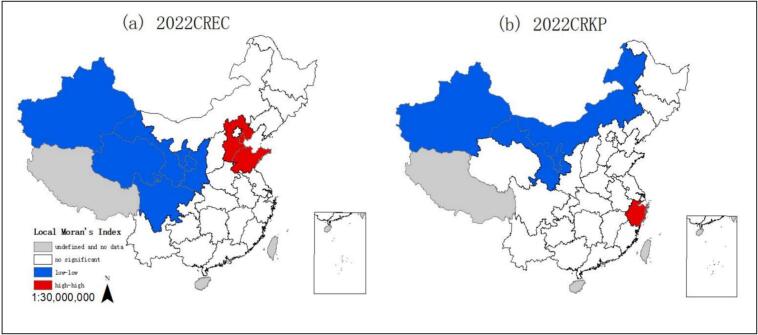
Local correlation LISA aggregation graph. Note: CREC: Carbapenem resistant *Escherichia coli*, CRKP: Carbapenem resistant *Klebsiella pneumoniae.*

During the study period, the Local Moran scatter diagrams demonstrated a distinct upward trend in the local Moran's *I* of CREC, whereas for CRKP, there was an initial decline followed by a subsequent increase. Many of the scatter points are concentrated in the first and third quadrants, representing High-High and Low-Low spatial clustering, which suggests a distinct spatial aggregation across provinces (Fig. S2 in Supplementary file). According to the 2022 LISA cluster map, the High-High cluster was found to take in Tianjin, Hebei and Shandong, indicating that CREC rate in these areas was relatively high and that the spatial correlation of CREC between regions was strong. Provinces with low CREC rate were principally centralized in Xinjiang, Gansu, Sichuan, Ningxia and Shaanxi, indicating that the spatial correlation of CREC between provinces was weak. For CRKP, the High-High cluster includes Zhejiang, which requires monitoring. The Low-Low cluster was found to take in Xinjiang, Gansu, Ningxia and Nei Mongol (Fig. 5).

### 3.4 Factors associated with CREC and CRKP rate

We found that SO_2_, NO_2_, CO and annual average humidity were mainly negatively correlated with CREC and CRKP rates. The average coefficients are −0.01399, −0.01941, −0.09188 and − 0.02225 respectively. While PM_2.5_, O_3_, annual average temperature and annual cumulative precipitation were mainly positively correlated with CREC and CRKP rates. The average coefficients are 0.00679, 0.03028, 0.02749 and 0.00045 respectively (Table 1).

**Table 1:** Descriptive analysis of independent variable coefficients.

Dependent variable	Independent variable	Mean	Max	Min	Lower Quartile	Median	Upper Quartile	SD
CREC	SO_2_	−0.01399	0.01811	−0.06525	−0.01654	−0.01178	−0.00671	0.01404
	NO_2_	−0.01941	0.11306	−0.05585	−0.03217	−0.02442	−0.01139	0.02099
	CO	−0.09188	1.77160	−0.49394	−0.34415	−0.20087	0.02684	0.37312
	O_3_	0.00679	0.10057	−0.01294	0.00491	0.00676	0.00832	0.00817
	PM_2.5_	0.03028	0.20714	−0.03461	0.02613	0.03194	0.03673	0.01849
	AMT	0.02749	0.48966	−0.09144	0.00039	0.01822	0.04006	0.05554
	AP	0.00045	0.01681	−0.00089	0.00022	0.00031	0.00042	0.00142
	ARH	−0.02225	0.02007	−0.63001	−0.02435	−0.01107	−0.00767	0.05518
CRKP	SO_2_	−0.20739	0.199071	−0.717306	−0.28854	−0.18736	−0.12277	0.16037
	NO_2_	−0.03139	0.459340	−0.411608	−0.13656	−0.06824	0.062005	0.16517
	CO	−1.42124	16.73275	−11.00659	−3.61555	−1.03794	−0.01575	3.52492
	O_3_	0.023742	0.17099	−0.131337	−0.00836	0.03379	0.05835	0.04971
	PM_2.5_	0.078402	0.32667	−0.44476	0.043722	0.09608	0.14593	0.11266
	AMT	0.12184	2.17903	−1.88383	−0.43878	0.16370	0.61906	0.77056
	AP	0.00126	0.00971	−0.00659	−0.00083	0.00141	0.00298	0.00273
	ARH	−0.12853	0.47131	−0.57571	−0.25677	−0.13710	−0.02418	0.21868

Note: CREC: Carbapenem-resistant *Escherichia coli*, CRKP:carbapenem-resistant *Klebsiella pneumoniae* AMT: annual mean temperature, AP: annual precipitation, ARH: average annual relative humidity.

#### 3.4.1 Time heterogeneity

**Fig. 6:**
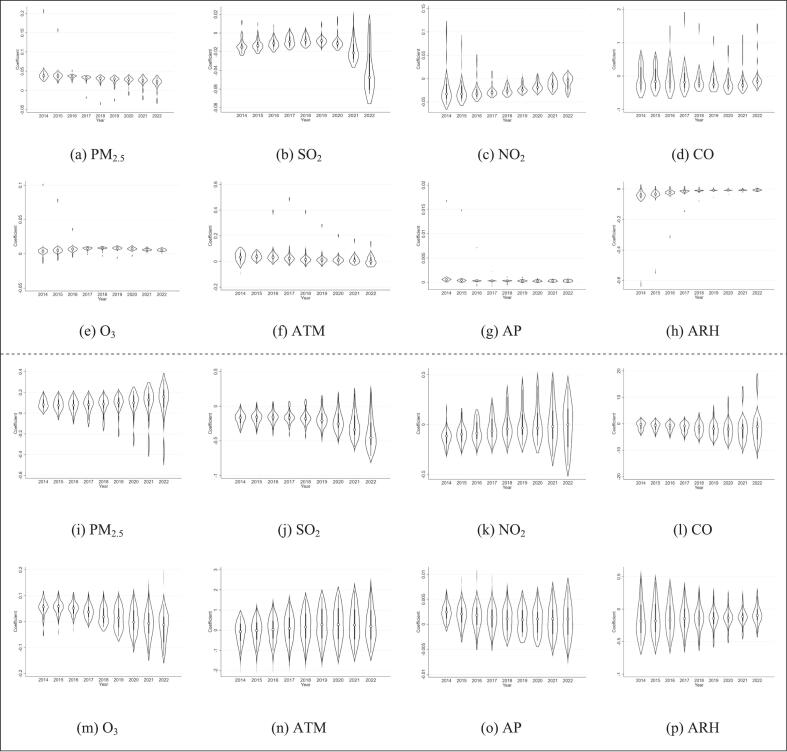
Temporal heterogeneity of influencing factors. Note: AMT: annual mean temperature, AP: annual precipitation, ARH: average annual relative humidity. CREC: (a)-(h),CRKP: (i)-(p).

During the research period, air pollution factors had different correlations with the rates of CREC and CRKP. PM_2.5_ and O_3_ are positively correlated with the CREC rate, and the trend is relatively stable. On the contrary, SO_2_, NO_2_ and CO are negatively correlated with the CREC rate. The impact of SO_2_ on CREC rate decreased between 2014 and 2018, followed by an increase during 2018 and 2022. Meanwhile, the impact of NO_2_ steadily declined, and the effects of CO remained relatively constant. In addition, PM_2.5_ positively contributed to the CRKP rate, with a persistent uptrend observed over the years. Both SO_2_ and CO show a negative correlation with the rate of CRKP, among which the influence of SO_2_ shows an increasing trend. The correlation between NO_2_ and CRKP rates changes from negative to positive, while O_3_ changes from positive to negative. (Fig. 6).

During the study period, climatic factors were significantly correlated with the rates of CREC and CRKP. The annual average temperature and the annual cumulative precipitation will increase the CREC rate, but the influence of the annual average temperature gradually weakens, and the influence of the annual cumulative precipitation remains stable at a relatively weak level. The annual average humidity is negatively correlated with the CREC rate, and its influence gradually weakens over time. In addition, the annual average temperature and the annual cumulative precipitation will increase the CRKP rate, and the influence of temperature is on the rise, while the influence of precipitation is relatively stable. The annual average humidity is negatively correlated with the CRKP rate, but this negative impact has been on the decline. (Fig. 6).

#### 3.4.2 Spatial heterogeneity

**Fig. 7:**
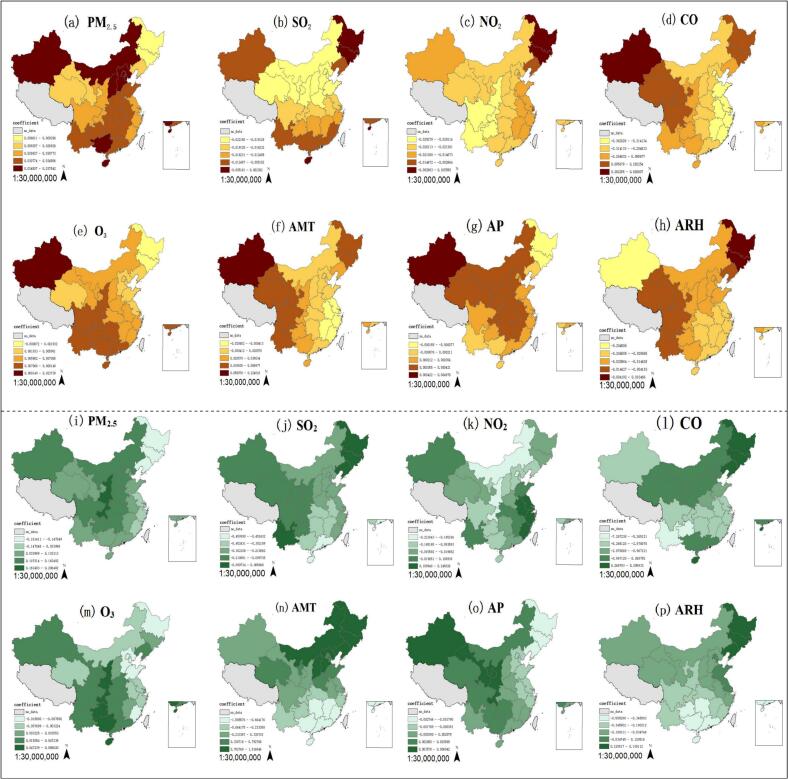
Spatial heterogeneity of influencing factors. Note: AMT: annual mean temperature, AP: annual precipitation, ARH: average annual relative humidity. CREC: (a)-(h),CRKP: (i)-(p).

PM_2.5_ and O_3_ are positively correlated with the CREC rates in different regions. PM_2.5_ has a significant impact on Xinjiang (coefficient = 0.038), Guangxi (coefficient = 0.035), Hainan (coefficient = 0.035) and North China (example: Hebei coefficient = 0.036). O_3_ has a significant impact on Xinjiang (coefficient = 0.024), the southwestern (example: Sichuan coefficient = 0.008) and southeastern coastal areas (example: Zhejiang coefficient = 0.006). SO_2_ and NO_2_ are mainly negatively correlated with the CREC rate. The influence of SO_2_ shows a trend of being lower in the south and higher in the north, gradually intensifying from south to north. The region's most closely associated with NO_2_ are mainly distributed in the southwest. CO is positively correlated with CREC in Heilongjiang (coefficient = 0.282), Jilin (coefficient = 0.163) and the western region (example: Gansu coefficient = 0.185), and negatively correlated with CREC in the eastern (example: Zhejiang coefficient = −0.362) and central regions (example: Jiangxi coefficient = −0.345). The annual average temperature will increase the CREC rate in most areas of China, with high value areas in western China (example: Yunnan coefficient = 0.045), Heilongjiang (coefficient = 0.059) and Jilin (coefficient = 0.044). However, it shows a negative correlation with the CREC rate in East China (example: Jiangsu coefficient = −0.004). Annual precipitation will increase the CREC rate, especially in Central (example: Jiangxi coefficient = 0.0004) and North (example: Beijing coefficient = 0.0003) China. The annual average relative humidity is mainly negatively correlated, with a greater correlation in the central (example: Henan coefficient = −0.020) and southeastern coastal cities (example: Jiangsu coefficient = −0.022). (Fig. 7).

PM_2.5_ and O_3_ mainly increase the rate of CRKP. However, PM_2.5_ is negatively correlated with CRKP in Northeast China (example: Heilongjiang coefficient = −0.147). O_3_ is negatively correlated with the CRKP rate in some provinces of North China (example: Hebei coefficient = −0.014). SO₂ is mainly negatively correlated with the CRKP rate, while it shows a positive correlation with the CRKP rate in Northeast China (example: Heilongjiang coefficient = 0.086). CO is mainly negatively correlated with the CRKP rate, but it exhibits a positive correlation with the CRKP rate in Northeast China (example: Heilongjiang coefficient = 5.296). NO_2_ can reduce the rate of CRKP, and the positive correlation is mainly concentrated in the East China region (example: Zhejiang coefficient = 0.205). The annual average temperature shows a positive effect in some areas of North China (example: Beijing coefficient = 0.793), Northeast China (example: Jilin coefficient = 1.268) and Northwest China (example: Gansu coefficient = 0.331), while it mainly shows a negative effect in East China (example: Fujian coefficient = −1.586) and Central South China (example: Hunan coefficient = −0.726). Annual precipitation will increase the CRKP rate in the western (example: Shanxi coefficient = 0.005) and central (example: Hubei coefficient = 0.002) and southern regions (example: Guangdong coefficient = 0.002), while showing a negative correlation with the northeastern region (example: Heilongjiang coefficient = −0.002) and most provinces along the eastern coast (example: Jiangsu coefficient = −0.001). The annual average relative humidity is mainly negatively correlated with the CRKP rate, and it gradually increases from north to south. (Fig. 7).

#### 3.4.3 Sensitivity analysis

The results of the sensitivity analysis have certain similarities with those of the GTWR model. The R^2^ of the four models ranges from 0.30 to 0.36. The explanatory power of the models is acceptable, but it is weaker than that of the GTWR model. Some variables lacked significance after incorporating three socio-economic control variables. For CREC, it was found from model2 that CO, annual mean temperature and annual relative humidity lacked significance after incorporating socio-economic variables, but their association directions were still consistent with the average values of the coefficients of each variable obtained from the GTWR model. Other environment variables were significantly in model 2, consistent with the results of GTWR model. Meanwhile, in model1, CO was significant at the level of *p* ≤ 0.1. For CRKP, it was found from model4 that NO_2_ and O_3_ lack significance after incorporating socio-economic variables, but their association directions still remain consistent with the average values of the coefficients of each variable obtained from the GTWR model. The other environment variables in Model 4 are consistent with the results of the GTWR model. Meanwhile, NO_2_ and O_3_ were significant in model3 at the p ≤ 0.1 and *p* ≤ 0.01 levels, respectively. (Table S4，Table S5 in Supplementary file and Table 1)

## 4 Discussion

Our study utilized the GTWR model for the first time to examine the relationship between antibiotic resistance rates (CREC and CRKP) and environmental factors (air pollution and climate factors), resulting in novel research findings. For air pollution factors, we found that PM_2.5_ and O_3_ mainly increase the rates of CREC and CRKP. However, SO_2_, NO_2_ and CO may reduce the rates of CREC and CRKP. In terms of climatic factors, the annual average humidity has a significant negative correlation with both CREC and CRKP rates, while the annual average temperature and the annual cumulative precipitation increase these two rates. Our study is also the first to investigate the spatial clustering of antibiotic resistance in China. Both CREC and CRKP displayed significant spatial aggregation, with High-High aggregation and Low-Low aggregation in local spatial autocorrelation. At the same time, our study also explains trends in antibiotic resistance over time and space. The trend analysis revealed that CREC and CRKP showed an upward trend in the east-west direction, and an inverted U-shape in the north-south direction. Furthermore, the CRKP rate exhibited an increasing trend over time.

The impact of PM_2.5_ on both CREC and CRKP is mainly positive, consistent across time and space. In terms of spatial variation, PM_2.5_ significantly affects regions with high rates of drug resistance. This aligns with the findings of previous research indicating that antibiotic resistance increases as PM_2.5_ levels rise [14]_._ The latest global study on climate change and antibiotic resistance also shows that PM2.5 is positively correlated with antibiotic resistance [35]. On a micro level, PM_2.5_ can promote the horizontal gene transfer of antibiotic resistance genes among bacteria. For instance, the efficiency of conjugative transfer of antibiotic resistance genes is enhanced by specific concentrations of PM_2.5_, which in turn upregulates the expression levels of genes associated with reactive oxygen species, the SOS response, cell membrane integrity, pilus formation, and transposition [36]. At the same time, a global study highlights that mitigating air pollution to lower PM_2·5_ concentrations could yield substantial health and economic benefits by diminishing antibiotic resistance [14]. A study in China also found a positive correlation between Antibiotic Resistance Genes and PM_2.5_ concentration, and noted a reduction in the prevalence of Antibiotic Resistance Genes as PM_2.5_ levels declined [37]. PM_2.5_ has been identified to contain a variety of antibiotic resistant bacteria and genes for antibiotic resistance, which can be transferred across diverse environments and directly inhaled by humans [14]. This exposure can lead to respiratory tract injuries and infections, and the subsequent treatment of these illnesses may promote increased use of antibiotics, further escalating drug resistance. This illustrates a potential crucial way that PM_2.5_ contributes to the intensification of antibiotic resistance.

The impact of SO_2_, NO_2_, and CO on the two bacterial strains was predominantly negative in terms of temporal heterogeneity. The previous study revealed that air with chemical pollution had fewer drug-resistant bacteria than particulate-polluted air, and resistance genes in the latter were more strongly correlated with pathogens, implying that airborne particulates more potently foster the coexistence of pathogens and resistance genes more than chemical pollutants [12]. Existing research suggests that these chemical air pollutants may affect the expression of drug resistance genes by altering the REDOX state of bacteria, potentially inhibiting the spread of drug resistance [[38], [39], [40]]. Although current research on the relationship between SO_2_, NO_2_, CO and antibiotic resistance is relatively limited. However, we still find that some studies suggest that when SO_2_, NO_2_ and CO reach a certain concentration, they can damage the biological macromolecules such as DNA, lipids and proteins of bacteria, disrupt the structure of bacteria, interfere with metabolism, enhance antibiotic sensitivity, and at the same time, do not induce drug resistance when repeatedly exposed, thus achieving effective sterilization and curbing the development of antibiotic resistance [41]. Regarding spatial impact, NO_2_ and CO have shown positive effects in certain areas, possibly due to increased respiratory diseases and tuberculosis associated with air pollution, which indirectly results in increased antibiotic use and subsequently contributes to the rise and spread of drug resistance [12].

On the contrary, the impact of O_3_ on the two bacterial strains appears to be predominantly positive across both temporal and spatial dimensions. Although existing studies indicate that O_3_ can be used as a bactericide, its efficacy in degrading bacteria requires specific concentrations and environmental conditions [[42], [43], [44]]. Simultaneously, O_3_ may promote the generation of antibiotic resistance when it reacts with drug-resistant bacteria [45]. However, research into the impact of such air pollutants on antibiotic resistance is limited, and this study is the first to investigate these effects at a macro level. Existing studies show that O_3_ can harm the human body, leading to conditions such as chronic obstructive pulmonary disease and cardiovascular disease [46,47]. This may also increase antibiotic use and contribute to drug resistance. More studies are needed to confirm these findings.

The impact of the annual mean temperature on CREC and CRKP was predominantly positive over time. Spatially, the annual mean temperature had a favorable effect on both strains, although it showed adverse effects in certain areas, particularly in the southern region, which is consistent with previous research. A review study on climate change and antibiotic resistance indicates that the local temperature rise caused by climate warming is associated with the increased spread and distribution of common *Escherichia coli* and *Klebsiella pneumoniae* [48]. Using data from the Korea Global Antimicrobial Resistance Surveillance System from 2017 to 2021, a previous study examined the relationship between local temperature and CRKP, it discovered that warm temperatures exacerbated the rate of CRKP [49]. Two studies conducted in China have determined that rising ambient temperatures increased antibiotic resistance, particularly in *E. coli* and *K. pneumoniae* to carbapenems [15,50]. Concurrently, the Chinese study also found that in southern China, temperature may adversely affect antibiotic resistance, potentially due to the region's relatively stable ambient temperature of 15–20 °C. From a microcosmic perspective, the connection between temperature and antibiotic resistance can be explained by their physiological similarities in triggering cell responses. Both aminoglycosides (a type of antibiotics) and heat stress enhance misfolded proteins in cells [51,52]. Consequently, heat exposure can stimulate bacteria to develop antibiotic resistance via a mechanism termed cross-tolerance [53,54].

Temporally and spatially, the effect of annual precipitation on both strains was positively and significantly correlated. However, the degree of influence observed in this study was minimal, with limited heterogeneity. Previous research has indicated that heightened precipitation leads to increased runoff, consequently resulting in elevated water pollution levels [55]. Resistant bacteria and resistant genes may accumulate in water pollution and may spread in a certain spatial range with runoff [55]. A global study has found a positive correlation between surface runoff and antibiotic resistance, and there is also a time lag [35]. The spatial-temporal effects of annual mean relative humidity on the two strains were mainly negative. Nevertheless, this impact exhibited a diminishing trend over time and approached zero. This finding is consistent with a global analysis of antibiotic resistance, which revealed that the average relative humidity had a negative impact on certain drug-resistant bacteria [21].Our findings are similar to those of a previous study on antibiotic resistance in 28 provinces in China, which found no significant association between annual precipitation, annual mean relative humidity and antibiotic resistance [15]. However, our study shows that the effects of precipitation and humidity on antibiotic resistance need to be equally valued [13]. The average concentration of culturable *E. coli* in storm drain outfalls during wet weather is ten times greater than that observed during dry weather [56]. There may be some synergistic effect between rainfall and humidity that has an impact on antibiotic resistance. More research is needed to confirm this.

In the sensitivity analysis, for CREC, we found that CO, annual mean temperature and annual relative humidity lacked significance after including socio-economic variables. For CRKP, we found that NO_2_ and O_3_ lacked significance after incorporating socioeconomic variables. On the one hand, this might be due to the inclusion of socio-economic variables, which dilutes their explanatory power. This situation is particularly common in spatial models, where variables in geographical space often have a common distribution trend [57] (such as the spatial coupling of natural environment variables and socio-economic variables). Controlling socio-economic variables essentially involves eliminating the common spatial trend to make the independent effect of the independent variables clearer. For instance, annual mean temperature, CO, NO_2_, O_3_ are all related to per capita GDP, medical infrastructure, etc. [[58], [59], [60]]. When these socio-economic variables are included, they may dilute the explanatory power of environmental variables. Some studies have found that the association between environmental temperature and antibiotic resistance is regulated by socioeconomic status. A higher economic status weakens the association between temperature and the detection rate of CRKP [61]. Some studies have also found that there is a certain correlation between the number of hospital beds and antibiotic resistance, which will weaken the association between environmental factors and antibiotic resistance [62]. Some studies have also found that there is no association between humidity and antibiotic resistance in the global regression model, which is consistent with the results of sensitivity analysis [62]. On the other hand, compared with the GTWR model, the spatial error model is a global regression model. The GTWR model can capture local regression results, that is, it can capture the direction and magnitude of the association between independent variables and dependent variables in different spaces and times, which is something that spatial error models cannot achieve [22]. The spatial error model assumes that the influence of environmental variables on the drug resistance rate is uniform throughout the region, but CREC and CRKP have local spatial aggregation, and there are also regional differences in environmental variables. GTWR can discover the significance of these variables through local regression. However, after the spatial error model averaged the data of the entire region, the local associations were diluted, which might eventually lead to some environmental variables no longer being significant in the sensitivity analysis. Meanwhile, the sensitivity analysis suggests that we need to be cautious about the correlations between CO, annual average temperature, annual relative humidity and CREC. The association among NO_2_, O_3_ and CRKP also needs to be treated with caution. In the future, the robustness of the correlation needs to be determined through further exploration at a smaller scale. At a small scale, the coupling degree between environmental variables and socio-economic variables is reduced, and it can match the micro-scenario of the spread of drug-resistant bacteria. Meanwhile, fine variables such as the rate of antibiotic prescriptions are introduced to further eliminate interference and ensure the robustness of the association.

The spatial trend analysis revealed that CREC and CRKP showed an upward trend in the east-west direction, while displaying an inverted U-shape pattern in the north-south direction. From an east-west perspective, our findings are consistent with existing studies, indicating a higher distribution of antibiotic resistance genes in China's eastern region compared to the western [63]. This discrepancy might be attributed to the greater availability medical resources in the eastern region, resulting in more prevalent antibiotic use and subsequently contributing to increased drug resistance [3]. From north to south, the inverted U pattern mirrors the outcomes of the current bacterial resistance monitoring network in China. Provinces in central China, especially Jiangxi, Anhui, Hunan and Henan, reported high CREC and CRKP rates, surpassing the national average. A comparative study across eastern, central and western China also found that third-generation cephalosporin-resistant *E. coli* manifested the highest resistance rate in the central region [3]. The spatial positive correlation between the rates of CREC and CRKP might be associated with regional variations in antibiotic resistance management policies and economic development [20]. Provinces in the same region may have more consistent economic development and antibiotic management policies, leading to spatial clustering [4]. On the other hand, antibiotic resistance genes can spread through environmental media, such as water and air [64]. The closer the geographical proximity, the easier it is for antibiotic resistance genes to transfer through the environment, leading to High-High and Low-Low. The observed rise in the CRKP rate over time aligns with previous findings indicating a global increased in CRKP rates [65].

From a One Health perspective, there exists a more intricate connection between AMR, air pollution, and climate change [11]. Air pollution and climate change can impact AMR through human, animal, and environment-related activities [11]. Public health departments, meteorological departments, environmental departments, etc. need to carry out cross-departmental cooperation to deal with the threat of drug resistance. We need to strengthen the monitoring of areas with high-high concentrations of antibiotic resistance detection rates across departments, and intervene in high-resistance areas such as eastern and central regions of China. Especially in small-scale regional areas, strengthen intervention measures. At the same time, regional collaborative governance should be carried out for PM_2.5_ and O_3_ as the main air pollutants, and climate change conditions such as temperature and rainfall should be incorporated into the comprehensive monitoring framework for the governance of antibiotic resistance. Incorporate indicators of antimicrobial resistance and climate change to strengthen the disease surveillance system.

This study had certain limitations. Firstly, factors such as the use of antibiotics and socio-economic conditions may also affect antibiotic resistance. However, this study only focused on the relationship between environmental variables and antibiotic resistance, which may lead to biased results. Previous studies have shown that regions with higher economic status may exhibit different patterns of antibiotic use (for example, higher consumption rates or prioritizing the use of specific antibiotic categories), which directly affects the development of antibiotic resistance [3,4]. At the same time, there are differences in environmental monitoring standards or pollution control measures, thereby introducing confusion into the relationship we are exploring. Although some studies on the impact of population density on antibiotic resistance have shown no significance, it may still affect the spread of drug-resistant bacteria to a certain extent [15]. Studies have shown that the more hospital beds there are per thousand people, the higher the drug resistance rate [15]. Population mobility and commodity trade are also associated with the spread of antibiotic resistance. Studies have shown that antibiotic resistance genes among the immigrant population are growing rapidly [66]. Studies have shown that commodity trading, such as some animal trading, may also carry the transfer of antibiotic resistance genes [67]. These unconsidered factors may lead to overestimation or underestimation of the true effect size. In future research, our goal is to address this limitation by integrating multi-source data on antibiotic use, socio-economic indicators, and medical policies, adopting more complex statistical models to unravel the intricate interactions among these factors, and enhancing the robustness of our conclusions.

Secondly, the potential synergistic effects between air pollutants and climate factors on antibiotic resistance necessitate further exploration. Finally, it is necessary to note that there may be ecological fallacies in the research. Meanwhile, due to the limitations of drug resistance data, the current research scale can only be measured at the provincial level. In the future, we will collect data from smaller-scale regions and conduct heterogeneity analysis within the province. Given that this study is the first to explore the relationship among air pollution, climatic factors and antibiotic resistance in China, further research is necessary.

## 5 Conclusion

In terms of air pollution, our research results show that PM_2.5_ and O_3_ are mainly positively correlated with CREC and CRKP rates. On the contrary, SO_2_, NO_2_ and CO may reduce these rates. In terms of climatic factors, the annual average humidity has a significant negative correlation with both CREC and CRKP, while the annual average temperature and the annual total precipitation have a significant positive correlation with both CREC and CRKP. Both CREC and CRKP exhibited significant spatial clustering, with areas of High-High and Low-Low clustering in local spatial autocorrelation analysis. The spatial analysis showed a rising pattern in the east-west direction and an inverted U-shape in the north-south direction for both CREC and CRKP rates. The study revealed an increasing trend in the CRKP rate across China, highlighting the necessity for ongoing monitoring and control measures. In summary, this study emphasizes the importance of ongoing attention to the impact of air pollution, climate factors, and other environmental changes on antibiotic resistance.

## CRediT authorship contribution statement

**Bingsong Li:** Writing – review & editing, Writing – original draft, Methodology, Formal analysis, Data curation, Conceptualization. **Xuemei Zhen:** Writing – review & editing, Supervision, Funding acquisition, Conceptualization. **Jiangfeng Ouyang:** Writing – review & editing. **Cecilia Stålsby Lundborg:** Writing – review & editing.

## Funding

This work was supported by 10.13039/501100001809National Natural Science Foundation of China (72304174), 10.13039/501100007129Natural Science Foundation of Shandong Province (ZR2025MS1150), Young Talent of Lifting engineering for Science and Technology in Shandong, China (SDAST2024QTA097) and the Open Fund of NHC Key Laboratory of Health Technology Assessment (Fudan University) of National Health Commission (FHTA2023-07).

## Declaration of competing interest

The authors declare that they have no known competing financial interests or personal relationships that could have appeared to influence the work reported in this paper.

## Data Availability

Data will be made available on request.

## Appendix A Supplementary data

Supplementary material Image 1

## References

[1] Tartari E. (2017). World Health Organization SAVE LIVES: clean your hands global campaign—‘fight antibiotic resistance—it’s in your hands’. Clin. Microbiol. Infect..

[2] Murray C.J.L. (2022). Global burden of bacterial antimicrobial resistance in 2019: a systematic analysis. Lancet.

[3] Zhen X. (2021). Socioeconomic factors contributing to antibiotic resistance in China: a panel data analysis. Antibiotics.

[4] Ahmad M., Khan A.U. (2019). Global economic impact of antibiotic resistance: a review. J. Glob. Antimicrob. Resist..

[5] Tacconelli E. (2018). Discovery, research, and development of new antibiotics: the WHO priority list of antibiotic-resistant bacteria and tuberculosis. Lancet Infect. Dis..

[6] Tomczyk S. (2019). Control of Carbapenem-resistant Enterobacteriaceae, *Acinetobacter baumannii*, and *Pseudomonas aeruginosa* in healthcare facilities: a systematic review and reanalysis of quasi-experimental studies. Clin. Infect. Dis..

[7] Gomez-Simmonds A. (2016). Evidence from a New York City hospital of rising incidence of genetically diverse carbapenem-resistant Enterobacter cloacaeand dominance of ST171, 2007–14. J. Antimicrob. Chemother..

[8] Savard P., Perl T.M. (2014). Combating the spread of carbapenemases in Enterobacteriaceae: a battle that infection prevention should not lose. Clin. Microbiol. Infect..

[9] Lin X.C. (2024). The global and regional prevalence of hospital-acquired Carbapenem-resistant *Klebsiella pneumoniae* infection: a systematic review and meta-analysis. Open Forum Infect. Dis..

[10] Kong H.F. (2024). Clinical risk factors and outcomes of carbapenem-resistant nosocomial infections in a Chinese teaching hospital: a retrospective study from 2013 to 2020. Microbiol. Spectr..

[11] Hernando-Amado S. (2019). Defining and combating antibiotic resistance from one health and Global Health perspectives. Nat. Microbiol..

[12] Gao M. (2023). Atmospheric antibiotic resistome driven by air pollutants. Sci. Total Environ..

[13] Burnham J.P. (2021). Climate change and antibiotic resistance: a deadly combination. Therap. Adv. Infect. Disease.

[14] Zhou Z. (2023). Association between particulate matter (PM)2·5 air pollution and clinical antibiotic resistance: a global analysis. Lancet Plan. Health.

[15] Li W. (2023). The Lancet Regional Health - Western Pacific.

[16] O’Mullan G.D., Juhl A., Young S. (2013). Antibiotic-resistant bacteria in the Hudson River estuary linked to wet weather sewage contamination. J. Water Health.

[17] Laroche E. (2010). Transport of antibiotic-resistant Escherichia coli in a public rural karst water supply. J. Hydrol..

[18] Xie J. (2018). Bacteria and antibiotic resistance genes (ARGs) in PM2.5 from China: implications for human exposure. Environ. Sci. Technol..

[19] Forrester J.D. (2022). Influence of socioeconomic and environmental determinants of health on human infection and colonization with antibiotic-resistant and antibiotic-associated pathogens: a scoping review. Surg. Infect..

[20] Hu F. (2018). Current status and trends of antibacterial resistance in China. Clin. Infect. Dis..

[21] Rahbe E. (2023). Determinants of worldwide antibiotic resistance dynamics across drug-bacterium pairs: a multivariable spatial-temporal analysis using ATLAS. Lancet Plan. Health.

[22] Huang B., Wu B., Barry M. (2010). Geographically and temporally weighted regression for modeling spatio-temporal variation in house prices. Int. J. Geogr. Inf. Sci..

[23] Liu Q. (2020). The varying driving forces of PM2.5 concentrations in Chinese cities: insights from a geographically and temporally weighted regression model. Environ. Int..

[24] Zhou C. (2024). Toward tuberculosis elimination by understanding epidemiologic characteristics and risk factors in Hainan Province, China. Infect. Dis. Pov..

[25] You J., Dong Z., Jiang H. (2024). Research on the spatiotemporal evolution and non-stationarity effect of urban carbon balance: evidence from representative cities in China. Environ. Res..

[26] Huang J. (2018). Health impact of China’s air pollution prevention and control action plan: an analysis of national air quality monitoring and mortality data. Lancet Plan. Health.

[27] Zhang L., Wei L., Fang Y. (2024). Spatial–temporal distribution patterns and influencing factors analysis of comorbidity prevalence of chronic diseases among middle-aged and elderly people in China: focusing on exposure to ambient fine particulate matter (PM2.5). BMC Public Health.

[28] He J. (2019). Exploring the regional differences of ecosystem health and its driving factors in China. Sci. Total Environ..

[29] Sharma A. (2024). Spatial analysis of food and water-borne diseases in Ahmedabad, India: implications for urban public health planning. Acta Trop..

[30] Chaiyana A. (2024). Leveraging remotely sensed and climatic data for improved crop yield prediction in the Chi Basin, Thailand. Sustainability.

[31] Balasubramani K. (2024). Spatio-temporal epidemiology and associated indicators of COVID-19 (wave-I and II) in India. Sci. Rep..

[32] Brunsdon C., Fotheringham S., Charlton M. (1998). Geographically weighted regression. J. Royal Stat. Soc. Ser. D (The Statistician).

[33] Dang H. (2025). Integrating geodetector and GTWR to unveil spatiotemporal heterogeneity in China’s Agricultural carbon emissions under the dual carbon goals. Agriculture-Basel.

[34] Guo B. (2021). Determining the effects of socioeconomic and environmental determinants on chronic obstructive pulmonary disease (COPD) mortality using geographically and temporally weighted regression model across Xi’an during 2014–2016. Sci. Total Environ..

[35] Li W.B. (2025). Changing climate and socioeconomic factors contribute to global antimicrobial resistance. Nat. Med..

[36] Xie S. (2019). The effect and mechanism of urban fine particulate matter (PM2.5) on horizontal transfer of plasmid-mediated antimicrobial resistance genes. Sci. Total Environ..

[37] Wang Q. (2023). Spatiotemporal dynamics, traceability analysis, and exposure risks of antibiotic resistance genes in PM2.5 in Handan, China. Environ. Sci. Pollut. Res..

[38] Lee G. (2024). Lab- and pilot-scale wet scrubber study on the redox-mediated simultaneous removal of NO and SO2 using a CaCO3-based slurry with KI as a redox catalyst. Chemosphere.

[39] Kondakova T. (2016). Response to gaseous NO2 air pollutant of *P. fluorescens* airborne strain MFAF76a and clinical strain MFN1032. Front. Microbiol..

[40] Pardeshi K.A. (2015). Thiol activated prodrugs of sulfur dioxide (SO2) as MRSA inhibitors. Bioorg. Med. Chem. Lett..

[41] Wang T.-Y., Zhu X.-Y., Wu F.-G. (2023). Antibacterial gas therapy: strategies, advances, and prospects. Bioact. Mater..

[42] Xu T. (2024). Progress in combating antibiotic resistance in animal agriculture. CyTA J. Food.

[43] Yoon Y. (2021). Degradation and deactivation of plasmid-encoded antibiotic resistance genes during exposure to ozone and chlorine. Water Res..

[44] Czekalski N. (2016). Inactivation of antibiotic resistant bacteria and resistance genes by ozone: from laboratory experiments to full-scale wastewater treatment. Environ. Sci. Technol..

[45] Keenum I. (2024). To what extent do water reuse treatments reduce antibiotic resistance indicators? A comparison of two full-scale systems. Water Res..

[46] Liang S. (2024). Long-term exposure to ambient ozone and cardiovascular diseases: evidence from two national cohort studies in China. J. Adv. Res..

[47] Xing Z. (2024). Combined effect of ozone and household air pollution on COPD in people aged less than 50 years old. Thorax.

[48] van Bavel B. (2024). Intersections between climate change and antimicrobial resistance: a systematic scoping review. Lancet Plan. Health.

[49] Yang J.W. (2024). Effect of temperature on Carbapenemase-encoding plasmid transfer in *Klebsiella pneumoniae*. Microorganisms.

[50] Li W. (2023). Estimating the effect of increasing ambient temperature on antimicrobial resistance in China: a nationwide ecological study with the difference-in-differences approach. Sci. Total Environ..

[51] Cardoso K. (2010). DnaK and GroEL are induced in response to antibiotic and heat shock in *Acinetobacter baumannii*. J. Med. Microbiol..

[52] Cruz-Loya M. (2019). Stressor interaction networks suggest antibiotic resistance co-opted from stress responses to temperature. ISME J..

[53] Andrade-Linares D.R., Lehmann A., Rillig M.C. (2016). Microbial stress priming: a meta-analysis. Environ. Microbiol..

[54] Hilker M. (2015). Priming and memory of stress responses in organisms lacking a nervous system. Biol. Rev..

[55] Yu W. (2023). An extensive assessment of seasonal rainfall on intracellular and extracellular antibiotic resistance genes in Urban River systems. J. Hazard. Mater..

[56] Ahmed W. (2018). Precipitation influences pathogenic bacteria and antibiotic resistance gene abundance in storm drain outfalls in coastal sub-tropical waters. Environ. Int..

[57] Zhang Z.W. (2023). Spatial-temporal heterogeneity of urbanization and ecosystem services in the Yellow River Basin. Sustainability.

[58] Zhang J.W. (2024). Exploring the modifying role of GDP and greenness on the short effect of air pollutants on respiratory hospitalization in Beijing. Geohealth.

[59] Asumadu-Sarkodie S., Owusu P.A. (2017). Carbon dioxide emissions, GDP per capita, industrialization and population: an evidence from Rwanda. Environ. Eng. Res..

[60] Berg K.A., Curtis C.C., Mark N.C. (2024). GDP and temperature: evidence on cross-country response heterogeneity. Eur. Econ. Rev..

[61] Zeng Y.C. (2023). The association between ambient temperature and antimicrobial resistance of in China: a difference-in-differences analysis. Frontiers. Public Health.

[62] Kou R.X. (2025). Panel data analysis of the effect of ambient temperature on antimicrobial resistance in China. Sci. Rep..

[63] Xu Z. (2023). Spatial distribution, pollution characteristics, and health risks of antibiotic resistance genes in China: a review. Environ. Chem. Lett..

[64] Jin L. (2021). Airborne transmission as an integral environmental dimension of antimicrobial resistance through the “one health” lens. Crit. Rev. Environ. Sci. Technol..

[65] Zhen X. (2020). Clinical and economic burden of Carbapenem-resistant infection or colonization caused by *Klebsiella pneumoniae*, *Pseudomonas aeruginosa*, *Acinetobacter baumannii*: a multicenter study in China. Antibiotics.

[66] Le Bastard Q. (2020). US immigration is associated with rapid and persistent acquisition of antibiotic resistance genes in the gut. Clin. Infect. Dis..

[67] Innes G.K. (2023). Distance and destination of retail meat alter multidrug resistant contamination in the United States food system. Sci. Rep..

