# Supplementary material for: Association between antibiotic resistance and air pollution and climate factors in China: A multivariable spatial-temporal analysis
Source: One Health. 2025 Sep 16;21:101204. doi: 10.1016/j.onehlt.2025.101204 (PMC12478133; doi:10.1016/j.onehlt.2025.101204)
Supplement: Supplementary file 1 — Supplementary material [file mmc1.docx]

Table S1 Description of the data used in this study

| Variable | Data definition |
| --- | --- |
| Carbapenem resistant *Escherichia coli* | *E. coli* bacteria develop resistance to carbapenem antibiotics (imipenem, meropenem or ertapenem) |
| Carbapenem resistant *Klebsiella pneumoniae* | *K. pneumoniae* bacteria develop resistance to carbapenem antibiotics (imipenem, meropenem or ertapenem) |
| PM_2.5_ | Annual average concentration of PM_2.5_ of major cities in each province/region (ug/m^3^) |
| PM_10_ | Annual average concentration of PM_10_ of major cities in each province/region (ug/m^3^) |
| O_3_ | Daily maximum 8-hour 90^th^ percentile concentration of O_3_ of major cities in each province/region (ug/m^3^) |
| SO_2_ | Annual average concentration of SO_2_ of major cities in each province/region (ug/m^3^) |
| NO_2_ | Annual average concentration of NO_2_ of major cities in each province/region (ug/m^3^) |
| CO | Daily average 95^th^ percentile concentration of CO of major cities in each province/region (mg/m^3^) |
| Temperature | Annual average temperature of major cities in each province/region (℃) |
| Precipitation | Annual cumulative precipitation of major cities in each province/region (mm) |
| Humidity | Annual average humidity of major cities in each province/region (%) |

| Table S2 Global Moran index | | | | | | | | | | |
| --- | --- | --- | --- | --- | --- | --- | --- | --- | --- | --- |
| Statistical value | | 2014 | 2015 | 2016 | 2017 | 2018 | 2019 | 2020 | 2021 | 2022 |
| Carbapenem resistant *Escherichia coli* | Moran’s I | 0.092 | -0.139 | 0.301 | 0.307 | 0.403 | 0.339 | 0.375 | 0.361 | 0.490 |
|  | Z | 1.006 | -0.892 | 2.617 | 2.776 | 3.445 | 2.988 | 3.210 | 3.172 | 4.222 |
|  | P | 0.163 | 0.198 | 0.012 | 0.008 | 0.004 | 0.005 | 0.005 | 0.002 | 0.001 |
| Carbapenem resistant *Klebsiella pneumoniae* | Moran’s I | 0.372 | 0.289 | 0.355 | 0.268 | 0.202 | 0.242 | 0.237 | 0.207 | 0.217 |
|  | Z | 3.260 | 2.548 | 3.082 | 2.452 | 1.976 | 2.306 | 2.208 | 1.958 | 2.041 |
|  | P | 0.002 | 0.012 | 0.008 | 0.018 | 0.033 | 0.024 | 0.026 | 0.041 | 0.033 |

| Table S3 Multicollinearity test based on OLS estimation | | | |
| --- | --- | --- | --- |
| Variable | VIF | 1/VIF | Mean VIF |
| SO_2_ | 2.52 | 0.396 | 5.24 |
| NO_2_ | 4.17 | 0.240 |  |
| PM_10_ | 12.96 | 0.077 |  |
| CO | 4.66 | 0.215 |  |
| O_3_ | 2.27 | 0.441 |  |
| PM_2.5_ | 7.64 | 0.131 |  |
| Annual mean temperature | 3.69 | 0.271 |  |
| Annual precipitation | 4.17 | 0.240 |  |
| Average annual relative humidity | 5.06 | 0.198 |  |

| Table S4 Parameters of four models | | | | | |
| --- | --- | --- | --- | --- | --- |
| Dependent variable | Model | OLS | TWR | GWR | GTWR |
| Carbapenem resistant *Escherichia coli* | AIC_c_ | 550.75 | 552.90 | 524.98 | 527.41 |
|  | R^2^ | 0.32 | 0.35 | 0.45 | 0.59 |
|  | bandwidth | - | 0.587 | 0.271 | 0.181 |
| Carbapenem resistant *Klebsiella pneumoniae* | AIC_c_ | 1720.99 | 1723.63 | 1630 | 1661.91 |
|  | R^2^ | 0.31 | 0.38 | 0.64 | 0.69 |
|  | bandwidth | - | 0.283 | 0.155 | 0.148 |

Table S5 Sensitivity analysis of spatial error model

| Variable | CREC | | | | CRKP | |
| --- | --- | --- | --- | --- | --- | --- |
|  | Model1  Coefficient（95%CI） | Model2  Coefficient（95%CI） | | Model3  Coefficient（95%CI） | | Model4  Coefficient（95%CI） |
| SO_2_ | -0.010***(-0.018,-0.002) | | -0.006*(-0.014,0.001) | -0.134***(-0.207,-0.061) | | -0.100***(-0.170,-0.029) |
| NO_2_ | -0.033***(-0.047,-0.018) | | -0.026***(-0.041,-0.011) | -0.108*(-0.243,0.028) | | -0.066(-0.202,-0.069) |
| CO | -0.187*(-0.396,0.022) | | -0.126(-0.327,0.075) | -3.136***(-5.097,-1.176) | | -2.656***(-4.497,-0.814) |
| O_3_ | 0.011***(0.006,0.017) | | 0.006**(0.000,0.011) | 0.074***(0.027,0.121) | | 0.021(-0.028,0.071) |
| PM_2.5_ | 0.038***(0.029,0.047) | | 0.035***(0.027,0.044) | 0.204***(0.119,0.290) | | 0.185***(0.103,0.267) |
| AMT | -0.009(-0.034,0.016) | | 0.007(-0.025,0.038) | 0.079(-0.160,0.317) | | 0.283**(0.000,0.565) |
| AP | 0.000***(0.000,0.001) | | 0.000***(0.000,0.001) | 0.003**(0.001,0.006) | | 0.002**(0.000,0.005) |
| ARH | -0.010(-0.025,0.004) | | -0.006(-0.022,0.010) | -0.166**(-0.303,-0.030) | | -0.176**(-0.324,-0.028) |
| LogGDP |  | | 0.770***(0.193,1.347) |  | | 12.846***(7.207,18.485) |
| Phy |  | | 0.026**(0.001,0.051) |  | | 0.027(-0.201,0.254) |
| Hb |  | | 0.011(-0.003,0.025) |  | | 0.193***(0.074,0.312) |
| R^2^ | 0.31 | | 0.30 | 0.30 | | 0.36 |

Note: CREC: Carbapenem resistant *Escherichia coli*, CRKP: Carbapenem resistant *Klebsiella pneumoniae*, AMT: annual mean temperature, AP: annual precipitation, ARH: average annual relative humidity, LogGDP: Logarithm of per capita GDP, Phy: Physicians per 10,000 population, Hb: Hospital beds per 10,000 population. ***indicates p≤0.01, **indicates p≤0.05 and *indicates p≤0.1.


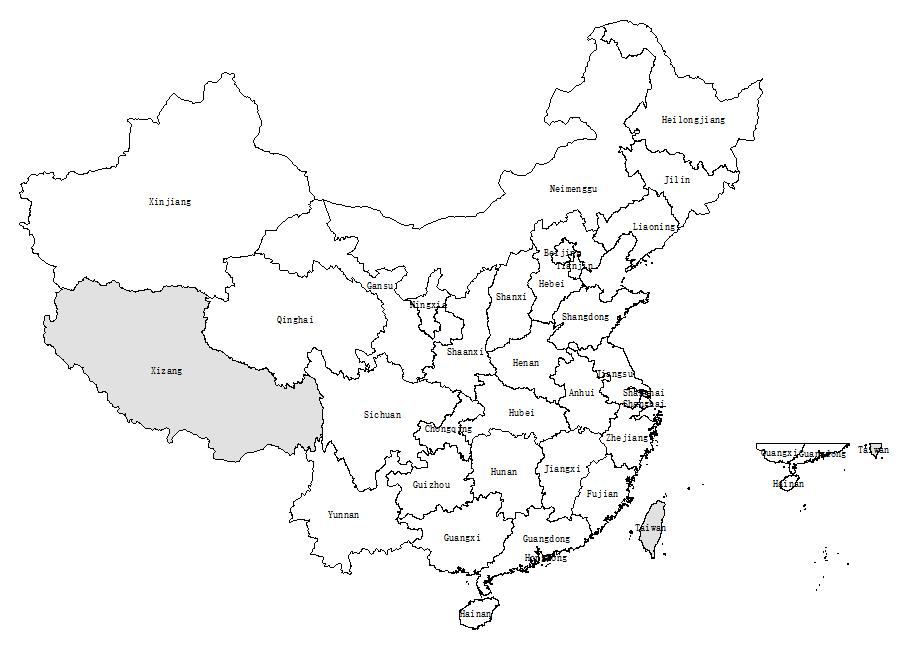


Figure S1 The names of provinces in China

Note: The gray part of four provinces (Taiwan, Hong Kong, Macau, and Tibet) was not included in the study.

| 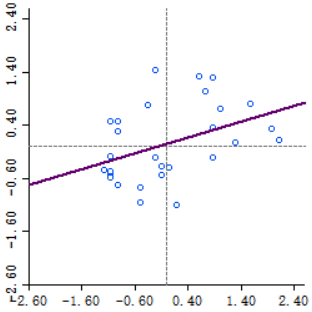  (a)2016 | 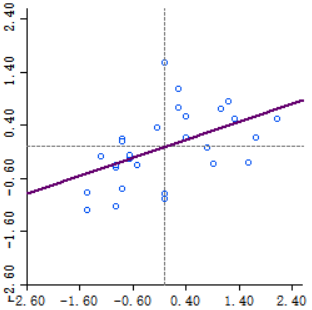  (b)2019 | 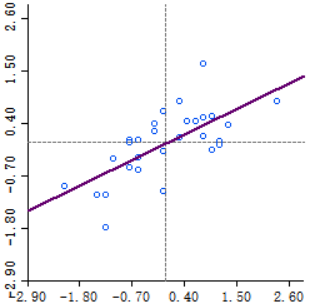  (c)2022 |
| --- | --- | --- |
| 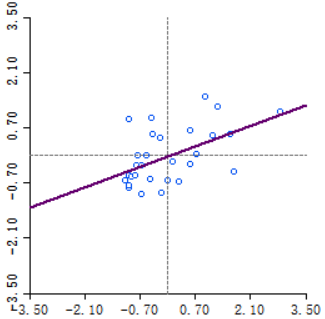  (d)2014 | 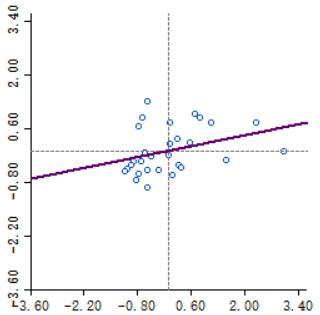  (e)2018 | 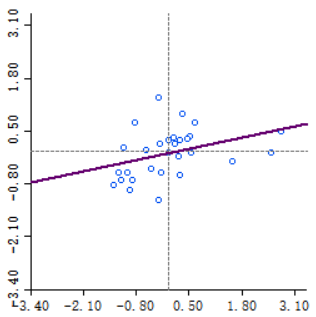  (f)2022 |
| Figure S2 Local correlation Moran scatter plot | | |
| Note:(a)(b)(c) showed Local Moran’s I index scatter plot of carbapenem-resistant *Escherichia coli*.(d)(e)(f) demonstrated Local Moran’s I index scatter plot of *Klebsiella pneumoniae* to carbapenems. | | |
